# Pre-Melting-Assisted Impurity Control of β-Ga_2_O_3_ Single Crystals in Edge-Defined Film-Fed Growth

**DOI:** 10.3390/nano15010007

**Published:** 2024-12-25

**Authors:** A-Ran Shin, Tae-Hun Gu, Yun-Ji Shin, Seong-Min Jeong, Heesoo Lee, Si-Young Bae

**Affiliations:** 1 Semiconductor Materials Center, Korea Institute of Ceramic Engineering and Technology, Jinju 52851, Republic of Korea; dkfks1217@pusan.ac.kr (A.-R.S.); shinyj@kicet.re.kr (Y.-J.S.); smjeong@kicet.re.kr (S.-M.J.); 2 School of Materials Science and Engineering, Pusan National University, Busan 46241, Republic of Korea; 3 Division of Nanotechnology and Semiconductor Engineering, Pukyong National University, Busan 49315, Republic of Korea; taehungu@pknu.ac.kr

**Keywords:** gallium oxide, impurity, pre-melting, EFG, single crystal

## Abstract

This study reveals the significant role of the pre-melting process in growing high-quality (100) β-Ga_2_O_3_ single crystals from 4N powder (99.995% purity) using the edge-defined film-fed growth (EFG) method. Among various bulk melt growth methods, the EFG method boasts a fast growth rate and the capability of growing multiple crystals simultaneously, thus offering high productivity. The pre-melting process notably enhanced the structural, optical, and electrical properties of the crystals by effectively eliminating impurities such as Si and Fe. Specifically, employing a 100% CO_2_ atmosphere during pre-melting proved to be highly effective, reducing impurity concentrations and carrier scattering, which resulted in a decreased carrier concentration and an increased electron mobility in the grown Ga_2_O_3_ single crystals. These results demonstrate that pre-melting is a crucial technique for substantially improving crystal quality, thereby promising better performance in β-Ga_2_O_3_-based device applications.

## 1. Introduction

Gallium oxide (Ga_2_O_3_) has attracted significant attention due to its exceptional properties, including an ultra-wide bandgap (4.5−5.3 eV), high breakdown field strength, a high critical electric field (8 MV/cm), and radiation hardness [1,2,3]. These characteristics make it highly suitable for high-power and high-temperature applications, such as power electronics for electric vehicles and renewable energy systems, including solar power inverters, wind turbine converters, and energy storage systems, where efficient power management is critical [4]. Among the five polymorphs of gallium oxide (Ga_2_O_3_)—α, β, γ, δ, and ε—the β phase is the only thermodynamically stable phase, making it the preferred choice for high-performance device applications [5,6,7,8]. While metastable polymorphs such as α-Ga_2_O_3_ (bandgap ~5.3 eV) and γ-Ga_2_O_3_ with unique photoluminescent properties are under investigation for specialized applications, their limited structural stability and growth challenges often make them less practical for large-scale device fabrication compared to β-Ga_2_O_3_ [7]. With a wide bandgap of ~4.8 eV, β-Ga_2_O_3_ exhibits a superior breakdown field strength when compared to classical semiconductors such as Si, Ge, and GaAs [9], as well as other technologically relevant wide-bandgap semiconductors such as SiC and GaN [10].

Recent advancements in Ga_2_O_3_ single-crystal growth have led to significant progress in developing large-diameter and high-quality Ga_2_O_3_ single-crystal substrates, driving the demand for more efficient and compact power devices [11]. Various growth techniques, such as Czochralski (CZ), floating zone (FZ), vertical Bridgman (VB), and edge-defined film-fed growth (EFG), have been employed to produce these substrates [12,13]. In particular, the EFG method offers several advantages for growing Ga_2_O_3_ single crystals compared to other techniques. It is well suited for producing large-diameter and high-quality substrates with controlled n-type doping profiles over a wide range from 10^15^ to 10^19^ cm^−3^ [14]. By utilizing a capillary die to shape the melt, EFG enables precise control over the crystal growth rate and dimensions [15]. Additionally, EFG offers a continuous growth process, which can lead to higher production rates and lower costs. These advantages make the EFG method a popular choice for manufacturing Ga_2_O_3_-based power devices.

The quality and properties of the Ga_2_O_3_ crystals grown using the EFG method are influenced by many process variables, including crystallographic orientation, growth processing factors, impurity incorporation, and others [16,17,18,19]. The choice of seed crystal orientation determines the crystallographic growth direction and the anisotropic properties of the grown material. The [010] direction is typically chosen for the pulling direction (i.e., growth direction) of β-Ga_2_O_3_ crystals in the EFG system. This orientation enables exposure to several primary planes of (001), (100), and (-201) depending on the rotation degree of seed crystals. The growth processing factors include temperature gradient, pull rate, atmosphere control phase equilibria, defect formation, and impurity incorporation [20]. The EFG crystal growth process commonly begins with seed touching, shaping (necking and shouldering), and body growth [21]. During this process, the large temperature gradient in the EFG system limits the process windows as the inclusion of polycrystals becomes prominent [22]. Among various gas atmospheres, CO_2_ is particularly effective in suppressing the thermal decomposition and volatilization of Ga_2_O_3_. This leads to a more uniform crystal structure and reduced defect formation compared to O_2_ or N_2_ atmospheres [19,20,22]. However, insufficient CO_2_ can destabilize IrO_2_, making it more likely to reduce to metallic Ir and evaporate. Conversely, excessive CO_2_ can lead to the formation of volatile IrO_3_ gas at high temperatures, also contributing to Ir loss during Ga_2_O_3_ pre-melting [23]. Therefore, finding the correct balance of oxygen partial pressure is crucial for minimizing Ir loss during crystal growth.

The quality of single crystals grown via the EFG method is highly sensitive to the presence of impurities, which can introduce various defects and negatively impact electrical and optical properties [24]. High-purity β-Ga_2_O_3_ single crystals might be obtained by employing high-purity raw materials (powders), suppressing contamination in equipment materials, and pre-annealing (or post-annealing) [14,25,26,27,28]. However, various environmental factors limit the purity of β-Ga_2_O_3_ single crystals, necessitating a classification of impurity types. Donor-like impurities (Sn, Si, Al, and Cr) create oxygen vacancies in Ga_2_O_3_, increasing electron concentration [29]. Conversely, acceptor-like impurities (Fe, Mg, and Ca) capture free electrons, reducing conductivity [30,31,32,33]. All these impurities should be minimized, except for intentional dopants (e.g., Sn or Si). Adopting high-purity materials in the EFG system is the best choice to obtain high-purity Ga_2_O_3_ crystals. However, inevitable factors during EFG operation, such as the use of low-purity (less than 4N) raw materials and contamination from equipment materials, can compromise the purity of Ga_2_O_3_ crystals. The use of high-purity materials alone is insufficient to mitigate these issues.

In this study, we investigate how the purity of Ga_2_O_3_ source material impacts the crystal quality, impurity incorporation, and electric characteristics of β-Ga_2_O_3_ single crystals grown using the edge-defined film-fed growth (EFG) method. Additionally, we propose using a pre-melting process to enhance the purity of low-purity Ga_2_O_3_ raw materials. Notably, pre-melting Ga_2_O_3_ raw materials necessitates a thorough consideration of thermodynamic process factors to minimize iridium volatilization, a common crucible material for Ga_2_O_3_ crystal growth. The purity of the bulk crystals was improved by reducing the contamination, originating from the powder material, and preventing reactions with the crucible, which decreases the free carrier concentration of the grown Ga_2_O_3_ bulk crystals and thus potentially could improve the Ga_2_O_3_-based electronic device performance.

## 2. Materials and Methods

For the starting raw materials, Ga_2_O_3_ powders with purities of 4N (Quantamaterials, Gyeongsan, Republic of Korea) and 5N (Lumi-M, Seoul, Republic of Korea) were prepared. A 2 mol% SnO_2_ dopant was used to grow n-type β-Ga_2_O_3_ crystals in the EFG system. The raw materials were placed in an Ir crucible within the EFG system and gradually heated to their melting point (1800 °C) using induction coil heating. During this process, the molten Ga_2_O_3_ ascended between the slits inside the crucible through capillary action. To suppress Ir loss, the growth atmosphere was maintained at 0.11 MPa with an Ar/CO_2_ gas ratio of 30:70. The β-Ga_2_O_3_ crystal was pulled at a rate of less than 15 mm/h along the [010] direction. After growth, the (100) β-Ga_2_O_3_ crystal was slowly cooled for approximately 15 h to room temperature. To elucidate the effect of pre-melting on raw materials, 4N Ga_2_O_3_ powder with a SnO_2_ dopant was melted at 0.11 MPa under various CO_2_ gas atmosphere contents ranging from 30% to 100% prior to crystal growth. Subsequently, pre-melted Ga_2_O_3_ with 100% CO_2_ was selected for crystal growth due to its lower impurity content. The structural properties of the grown (100) β-Ga_2_O_3_ single crystals were evaluated using scanning electron microscopy (SEM, JSM-6701F, JEOL, Tokyo, Japan). Elemental composition analysis of the grown samples was performed using glow discharge mass spectrometry (GDMS, VG9000, Eurofins, Fort Lauderdale, FL, USA). X-ray diffraction (XRD, Smartlab, Rigaku, Tokyo, Japan) was employed to assess crystal orientation and quality. Optical properties, including transmittance and optical bandgap, were analyzed using UV–visible spectroscopy (UV–Vis, Cary5000, Agilent, Santa Clara, CA, USA). Finally, Hall effect measurements (HMS-5300, ECOPIA, Toronto, ON, Canada) were conducted to determine the carrier concentration and mobility of the grown Ga_2_O_3_ crystals.

## 3. Results and Discussion

### 3.1. Effect of Powder Purity on β-Ga_2_O_3_ Crystal Growth

**Figure 1:**
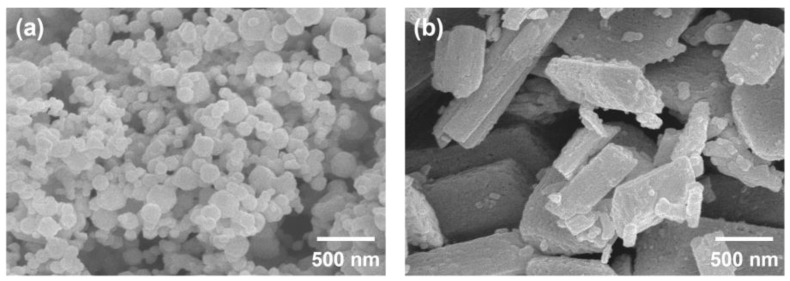
SEM images of (**a**) 4N and (**b**) 5N β-Ga_2_O_3_ powders.

Two types of Ga_2_O_3_ powders with different purities (4N and 5N) were prepared as starting materials for β-Ga_2_O_3_ crystal growth. Figure 1 shows the SEM images of the 4N and 5N β-Ga_2_O_3_ powders. 4N Ga_2_O_3_ powders were produced by thermal evaporation and exhibit a typical spherical nanoparticle morphology, as shown in Figure 1a [34]. In contrast, the 5N Ga_2_O_3_ powder, fabricated via a wet chemical process, displays a microflake morphology, as shown in Figure 1b [35]. These morphological differences can influence the volumetric filling ratio in the crucible of the crystal growth system and the impurity mixing ratio.

**Figure 2:**
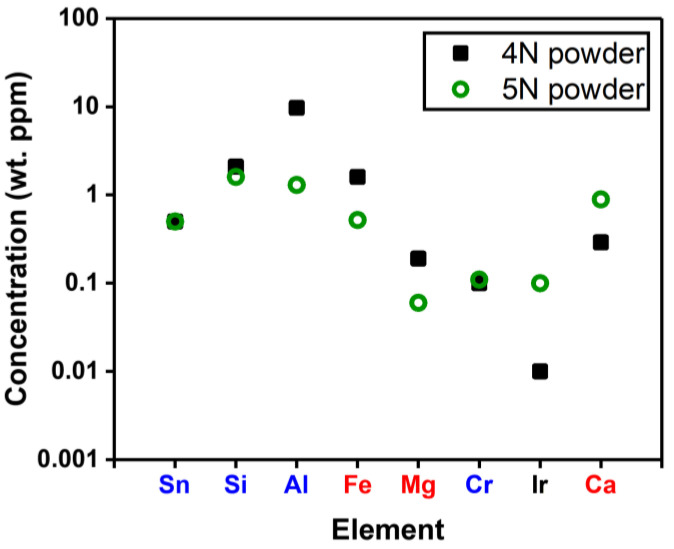
GDMS analysis of impurity composition in 4N and 5N β-Ga_2_O_3_ powders. Donor-like impurities (Sn, Si, Al, and Cr, marked in blue) and acceptor-like impurities (Fe, Mg, and Ca, marked in red) are identified.

Figure 2 compares the majority of impurities of 4N and 5N β-Ga_2_O_3_ powders measured via GDMS. Donor-like impurities (Sn, Si, Al, and Cr), marked in blue, are primarily substituted into Ga sites, acting as donors [32,36]. The Al in 4N powder, present in high concentrations, might form a ternary solid solution and lead to oxygen vacancies in β-Ga_2_O_3_ [37]. While an appropriate concentration of a donor such as Si can enhance electron transport, excessive Si leads to segregation and polycrystal formation [38]. Due to the similar atomic radii of Cr (0.062 nm) and Ga (0.062 nm), Cr impurities do not significantly affect the bandgap or structural deformation of β-Ga_2_O_3_. However, changes in photoluminescence in the visible to near-infrared range have been observed [39].

On the other hand, other impurities of Fe, Mg, and Ca, marked in red, can be categorized as deep-level acceptors in β-Ga_2_O_3_ [32,40,41]. Fe and Mg have an activation energy of ~0.86 eV and ~1.1 eV, respectively, reducing the conductivity and carrier mobility by capturing free electrons [32,33]. Ca has an activation energy of ~1.3 eV, forming a deep acceptor-like level that can act as a charge trap [42]. Overall, relatively high levels of impurities (>1 wt. ppm) such as Al, Si, and Fe are significant factors affecting the growth of β-Ga_2_O_3_ crystals. The high Al content in 4N Ga_2_O_3_ powder may originate from the use of alumina in the high-temperature evaporation method system.

**Figure 3:**
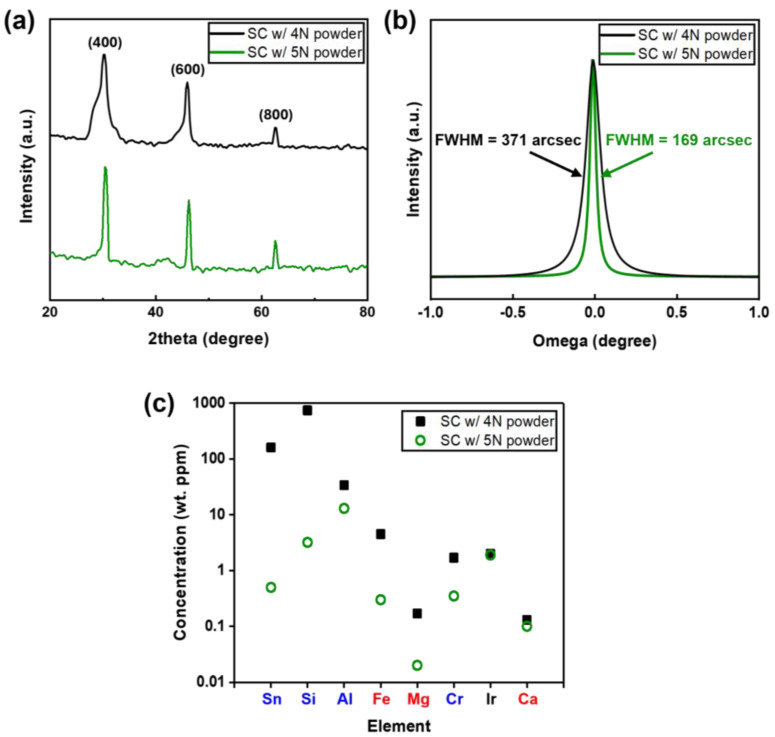
(**a**) XRD 2θ-scan spectra of (100) β-Ga_2_O_3_ single crystals grown from 4N and 5N powders. (**b**) XRD omega-scan spectra for the (400) diffraction peak. (**c**) Impurity concentrations measured by GDMS for (100) β-Ga_2_O_3_ grown from 4N and 5N powders.

Figure 3a,b show the XRD spectra of the β-Ga_2_O_3_ single crystals (SC) grown from 4N and 5N powders, respectively. As the (100) planes of β-Ga_2_O_3_ were the primary growth planes in the EFG process, the surfaces of all grown β-Ga_2_O_3_ single crystals were also the (100) plane, as shown in Figure 3a. The full width at half maximum (FWHM) of the (400) diffraction peak was 371 arcsec for crystals grown from 4N powder and 169 arcsec for those grown from 5N powder, as shown in Figure 3b. Hence, the crystal quality was superior for crystals grown from 5N powder. Figure 3c compares the concentrations of impurities in β-Ga_2_O_3_ crystals grown from 4N and 5N powders. The Sn dopant was more volatile in high-purity 5N Ga_2_O_3_ powder than in lower-purity 4N Ga_2_O_3_ powder. As Sn starts to evaporate at very high temperatures (>900 °C), high concentrations of Sn were distributed in the upper region of the Ga_2_O_3_ melt during crystal growth, while concentrations are lower in the lower region of the melt [43]. Additionally, Ga vacancies formed at high temperatures can readily capture Sn atoms, resulting in the formation of an energetically stable complex (V_Ga_Sn_Ga_, 1.63 eV), allowing Sn to either diffuse within the (100) β-Ga_2_O_3_ crystal or remain adsorbed on its surface [44]. The concentrations of Si and Al were significantly higher than expected. These impurities likely originated from silica, alumina, and other experimental equipment components [25]. In particular, the Ir crucible and alumina rod used in the EFG process are major sources of impurities. These impurities include elements such as Si, Al, Fe, Mg, Cr, Ir, and Ca, and in single crystals grown using 4N powder, the concentrations of Al and Si can exceed 10 wt. ppm. Additionally, the concentrations of element impurities like Si and Fe may increase due to the surrounding environment or source material during the single-crystal growth process. Therefore, the choice of starting material purity and experimental equipment is crucial for improving the quality of (100) β-Ga_2_O_3_ crystals.

### 3.2. Effect of Pre-Melting Process on β-Ga_2_O_3_ Crystal Growth

**Figure 4:**
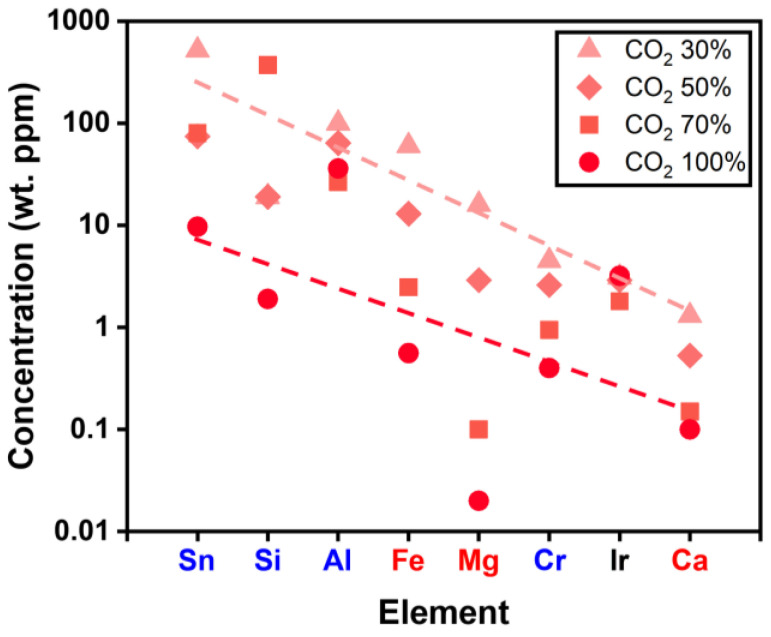
Impurity concentration of Ga_2_O_3_ pre-melt after the power heating process with various CO_2_ gas ratios (30–100%), measured by GDMS.

Although high-purity 5N Ga_2_O_3_ powder proved effective for growing high-quality Ga_2_O_3_ crystals, further purification of the starting materials is desirable. As mentioned earlier, the pre-melting process may be suitable for material purification. Here, we chose low-purity 4N powder for the pre-melting process, as impurity contamination from the EFG system is unavoidable unless the components are replaced. A pre-melting process was performed on 4N β-Ga_2_O_3_ powder to investigate the effect of various CO_2_ gas ratios on impurity concentration. CO_2_ is particularly effective in suppressing the thermal decomposition and volatilization of Ga_2_O_3_, leading to a more uniform crystal structure and reduced defect formation compared to O_2_ or N_2_ atmospheres [19,20,22]. Figure 4 shows the GDMS results of the pre-melt Ga_2_O_3_ with various CO_2_ gas ratios (30−100%), with elements sorted by decreasing concentration. Notably, as the CO_2_ concentration increases, the overall concentration of most elemental impurities tends to decrease.

The decrease in impurity concentrations can be attributed to the enhanced oxidation of impurities with an increasing CO_2_ ratio [45]. Most species are readily volatile after the pre-melting process, with the exception of Al. A careful analysis of the Ir concentration in the Ga_2_O_3_ pre-melt revealed that it was 3.1, 2.9, 1.8, and 3.2 wt. ppm at 30%, 50%, 70%, and 100% CO_2_, respectively. At a low CO_2_ ratio of 30%, oxygen availability is limited, leading to IrO_2_ instability and its potential reduction to metallic Ir, resulting in increased Ir loss [23]. On the other hand, at a high CO_2_ ratio of 100%, excessive oxygen promotes the formation of volatile IrO_3_ gas at elevated temperatures, leading to a similar increase in Ir content in the Ga_2_O_3_ pre-melt [23]. These results indicate that both insufficient and excessive oxygen can lead to significant Ir loss. Therefore, a 100% CO_2_ concentration should only be used in the pre-melting process, while a 70% CO_2_ concentration should be employed for Ga_2_O_3_ crystal growth to minimize Ir loss.

**Figure 5:**
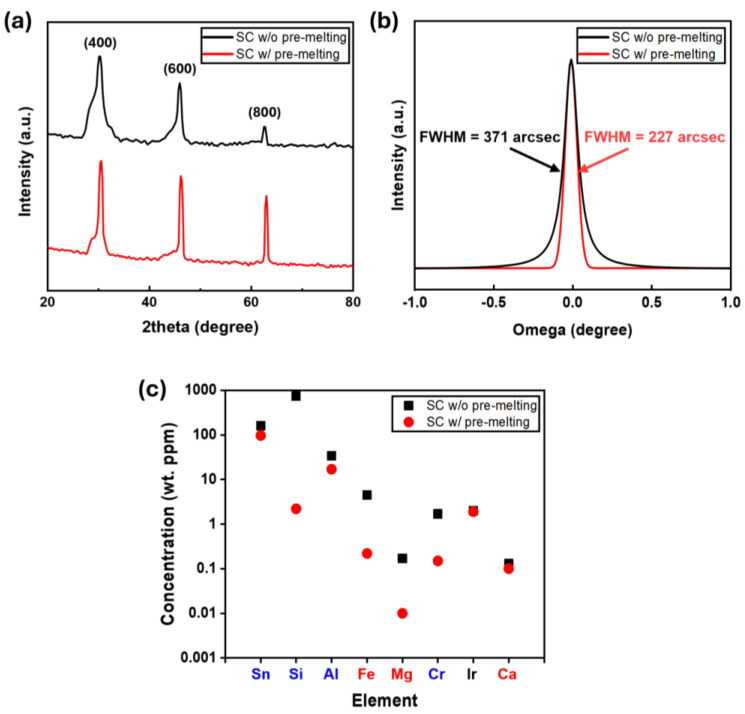
(**a**) XRD 2θ-scan spectra of (100) β-Ga_2_O_3_ single crystals grown with and without the pre-melting process. (**b**) XRD omega-scan spectra for the (400) diffraction peak. (**c**) Impurity concentrations measured by GDMS for (100) β-Ga_2_O_3_ grown with and without the pre-melting process.

Figure 5a,b show the XRD spectra of β-Ga_2_O_3_ single crystals grown with and without the pre-melting process. A combination of 100% CO_2_ gas during pre-melting and 70% CO_2_ gas during crystal growth was used to reduce impurities and maintain a sufficient oxidizing atmosphere. The (100) planes of β-Ga_2_O_3_ were observed for all grown β-Ga_2_O_3_ single crystals, as shown in Figure 5a. The FWHM of the (400) diffraction peak was 227 arcsec for crystals grown with pre-melting and 371 arcsec for those grown without pre-melting, as shown in Figure 5b. This indicates that pre-melting significantly improved crystal quality for low-purity Ga_2_O_3_ powder. It has been reported that the FWHM of the wafer-sliced (100) β-Ga_2_O_3_ single crystals was 59.4 arcsec [24]. Hence, the FWHM value of the grown (100) β-Ga_2_O_3_ single crystals might be further improved using the growth process optimization. Figure 5c compares the impurity concentrations of (100) β-Ga_2_O_3_ single crystals grown with and without pre-melting, as measured by GDMS. Several impurity elements (Si, Fe, Mg, and Cr) were reduced by an order of magnitude after pre-melting. The intentional Sn dopant was measured at a similar concentration level of ~100 wt. ppm in both β-Ga_2_O_3_ crystals. This is likely due to the reabsorption of Sn in the grown crystals, as Sn is quite volatile at high temperatures [44]. Impurities like Al and Ir, originating from the EFG system components, remained at similar concentrations in both samples.

### 3.3. Optical and Electrical Properties

**Figure 6:**
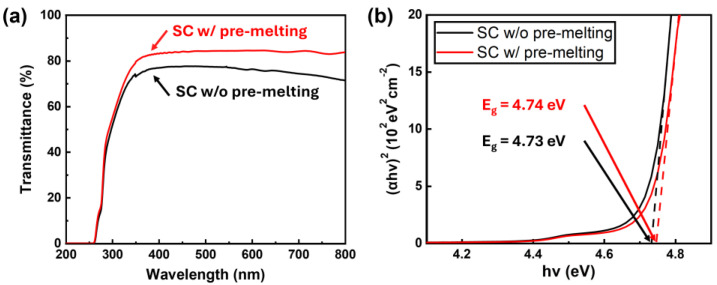
(**a**) UV/vis transmittance spectra of β-Ga_2_O_3_ single crystals grown with and without pre-melting and (**b**) the Tauc plot for determining the optical bandgap.

Figure 6a shows the UV−Vis transmittance spectra of β-Ga_2_O_3_ single crystals grown with and without pre-melting. Above a wavelength of 300 nm, the pre-melted sample exhibited higher transmittance of over 80%, approximately 5% higher than the sample without pre-melting. This increased transmittance can be attributed to reduced optical scattering caused by decreased impurity concentration in the pre-melted (100) Ga_2_O_3_ crystals [46]. The optical bandgap of each sample was analyzed using the Tauc plot method, as shown in Figure 6b. The bandgap energy is estimated through this method by plotting (αhν)² as a function of photon energy (hν) [47,48,49,50]. The bandgap of the pre-melted and non-pre-melted (100) Ga_2_O_3_ was determined to be 4.74 eV and 4.73 eV, respectively. The slight difference of 0.01 eV might be related to bandgap narrowing depending on the impurity concentration [51,52]. When impurities are abundant, the intrinsic bandgap might be distorted by changing the density of states. In particular, the pre-melting process significantly reduced the concentrations of Si, Fe, Mg, and Cr, as shown in Figure 5c. This reduction in impurities mitigated bandgap narrowing effects, leading to a recovery of the bandgap in (100) β-Ga_2_O_3_ crystals. Consequently, the pre-melting process effectively controlled impurity concentrations within the (100) β-Ga_2_O_3_ crystals, resulting in enhanced transmittance and an increased optical bandgap.

The electrical properties of the samples were characterized by Hall measurements, comparing carrier concentration, mobility, and resistivity, as shown in Table 1. The (100) Ga_2_O_3_ single crystal grown without pre-melting exhibited a low mobility of 58 cm²/V·s at a carrier concentration of 8.1 × 10^18^ cm^−3^. In contrast, the (100) Ga_2_O_3_ single crystal grown with pre-melting showed an increased mobility of 79.1 cm²/V·s at a carrier concentration of 3.5 × 10^18^ cm^−3^. The carrier concentrations from the C−V measurements were 1.7 × 10^19^ cm^−3^ and 1.0 × 10^18^ cm^−3^ for the SC without pre-melting and SC with pre-melting, respectively, identifying almost similar carrier concentrations. Sn was intentionally chosen as a dopant due to its favorable properties and stability under the given growth conditions [12]. However, the GDMS analysis revealed a high concentration of unintentional Si impurities in (100) Ga_2_O_3_ single crystals grown from low-purity starting materials. Insufficient CO_2_ gas ratios during growth can lead to Si accumulation [53]. The pure CO_2_ atmosphere (100% CO_2_ gas) applied during the pre-melting process effectively reduced Si concentration, which was subsequently maintained in the grown (100) Ga_2_O_3_ single crystals. This reduction contributed to improved electron mobility with suppressed ionized impurity scattering. Regarding resistivity, the sample without pre-melting exhibited a lower value of 1.3 × 10^−2^ Ω·cm, while the pre-melted sample showed a higher resistivity of 2.3 × 10^−2^ Ω·cm. The increased resistivity can be attributed to the reduced carrier concentration in the pre-melted (100) Ga_2_O_3_ sample. The Hall measurement results are consistent with the electrical properties of (100) Ga_2_O_3_ bulk crystals reported in the literature [12]. Overall, the pre-melting process effectively improved the optical and electrical properties of (100) Ga_2_O_3_ single crystals by reducing impurity concentrations. The availability of high-quality (100) Ga_2_O_3_ substrates has opened up new avenues for research and development in the field of wide-bandgap semiconductors, enabling the creation of devices capable of operating under harsh conditions and with improved efficiency.

## 4. Conclusions

The purity of starting materials was critical for growing high-quality single Ga_2_O_3_ crystals in the EFG system. Using 5N powders (99.999% purity) produced significantly better crystal quality compared to the 4N powders (99.995% purity). Lower-purity 4N powders were pre-melted under various CO_2_ gas ratios (30−100%) in the EFG system. A 100% CO_2_ gas ratio proved most effective in reducing impurities, leading to improved crystal quality. Impurities such as Al, Ir, and Ca, originating from the component materials in the EFG system, remained largely unchanged despite the purification process. In contrast, the pre-melting process effectively reduced impurities such as Si, Fe, Mg, and Cr, which significantly improved the properties of the (100) β-Ga_2_O_3_ crystals. The pure CO_2_ atmosphere effectively reduced the particular impurities, while Ir loss due to oxidation could be detrimental. In this sense, reducing the pre-melting process time might be a cost-effective approach. If impurities from the equipment apparatus could be suppressed, further purification could be obtained for the high-purity (>5N) powders. Therefore, pre-melting with a controlled CO_2_ gas ratio is a highly effective method for improving the characteristics of single β-Ga_2_O_3_ crystals, enabling the development of high-performance wide-bandgap semiconductors.

## Figures and Tables

**Table 1:** Comparison of material properties for β-Ga_2_O_3_ single crystals with and without the pre-melting process obtained via Hall and C−V measurements.

Property	Without Pre-Melting	With Pre-Melting	Remark
Carrier concentration (cm^−3^)	8.1 × 10^18^	3.5 × 10^18^	Hall
	1.7 × 10^19^	1.0 × 10^18^	C−V
Mobility (cm^2^/V·s)	58	79.1	Hall
Resistivity (Ω·cm)	1.3 × 10^−2^	2.3 × 10^−2^	Hall

## Data Availability

Data are contained within the article.
